# PD-1 Blockade Promotes Emerging Checkpoint Inhibitors in Enhancing T Cell Responses to Allogeneic Dendritic Cells

**DOI:** 10.3389/fimmu.2017.00572

**Published:** 2017-05-22

**Authors:** Carmen Stecher, Claire Battin, Judith Leitner, Markus Zettl, Katharina Grabmeier-Pfistershammer, Christoph Höller, Gerhard J. Zlabinger, Peter Steinberger

**Affiliations:** ^1^Division of Immune Receptors and T Cell Activation, Institute of Immunology, Center for Pathophysiology, Infectiology and Immunology, Medical University of Vienna, Vienna, Austria; ^2^Cancer Immunology and Immune Modulation, Boehringer Ingelheim RCV GmbH & CoKG, Vienna, Austria; ^3^Division of Clinical and Experimental Immunology, Institute of Immunology, Center for Pathophysiology, Infectiology and Immunology, Medical University of Vienna, Vienna, Austria; ^4^Department of Dermatology, Medical University of Vienna, Vienna, Austria

**Keywords:** PD-1, TIM-3, BTLA, CD160, LAG-3, CTLA-4, immune checkpoint, coinhibitory receptors

## Abstract

Immune checkpoint inhibitors, which target coinhibitory T cell molecules to promote anticancer immune responses, are on the rise to become a new pillar of cancer therapy. However, current immune checkpoint-based therapies are successful only in a subset of patients and acquired resistances pose additional challenges. Finding new targets and combining checkpoint inhibitors might help to overcome these limitations. In this study, human T cells stimulated with allogeneic dendritic cells (DCs) were used to compare immune checkpoint inhibitors targeting TIM-3, BTLA, LAG-3, CTLA-4, and TIGIT alone or in combination with a PD-1 antibody. We found that PD-1 blockade bears a unique potency to enhance T cell proliferation and cytokine production. Other checkpoint inhibitors failed to significantly augment T cell responses when used alone. However, antibodies to TIM-3, BTLA, LAG-3, and CTLA-4 enhanced T cell proliferation in presence of a PD-1 antibody. Upregulation of coinhibitory T cell receptors upon PD-1 blockade was identified as a potential mechanism for synergistic effects between checkpoint inhibitors. Donor-specific variation in response to immune checkpoint inhibitors was attributed to the T cells rather than DCs. Additionally, we analyzed the regulation of checkpoint molecules and their ligands on T cells and allogeneic DCs in coculture, which suggested a PD-1 blockade-dependent crosstalk between T cells and APC. Our results indicate that several immune checkpoint inhibitors have the capacity to enhance T cell responses when combined with PD-1 blockade. Additional *in vitro* studies on human T cells will be useful to identify antibody combinations with the potential to augment T cell responses in cancer patients.

## Introduction

Researchers and clinicians have attempted to harness the immune system to fight and eradicate tumor cells for well over 100 years (1, 2). However, broad clinical success has been achieved only recently with the advent of efficient blocking antibodies to T cell coinhibitory pathways–immune checkpoint inhibitors.

CTLA-4 was the first immune checkpoint that was successfully targeted with the CTLA-4 mAb ipilimumab in heavily pretreated melanoma patients, and this treatment prolonged overall median survival in two independent studies (3, 4). Importantly, clinical responses proved to be durable and survival benefit was observed across all lines of therapies, treatment regimens, and dose levels (5). Ipilimumab was approved in the US for the treatment of melanoma and is currently undergoing clinical trials for the treatment of other malignancies including lung cancer, pancreatic cancer, and metastasizing prostate cancer (6–8). PD-1 antibodies have more recently been shown to be highly active in various cancers including melanoma, non-small-cell lung cancers, Merkel-cell carcinoma, and renal cancer (9, 10). The combined use of PD-1 and CTLA-4 antagonists appears to have enhanced clinical efficacy compared to monotherapy with PD-1 or CTLA-4 antibodies (11). Thus, it is possible that antibodies targeting novel immune checkpoints will also enhance the antitumor activity of PD-1 antagonists. T cells express several additional coinhibitory receptors (12) and targeting these novel immune checkpoints alone or in combination with PD-1 may have potential in cancer therapy. Murine models are widely used to explore both the biology of these molecules and the efficacy of their antagonists to enhance T cell immunity (13–16). Moreover, several studies that have analyzed the function of blocking antibodies targeting novel immune checkpoints in human T cell responses *in vitro* have provided rationales for the therapeutic use of these checkpoint inhibitors (17–21). Nevertheless, there clearly is paucity in the data on immune checkpoint functions in human T cells. Few studies have compared several different immune checkpoints and in addition there is limited information regarding synergies and redundancies in the use of PD-1 blockers and immune checkpoint inhibitors targeting other coinhibitory T cell pathways.

Dendritic cells (DCs) are key regulators of immunity and thus also have an essential role in the initiation of T cell responses toward tumors (22). DC subsets endowed with the capacity to cross-present antigens efficiently prime tumor-specific CD8 T cells for the differentiation into CTLs that eradicate malignancies (23). Importantly, the immune checkpoints are not confined to T cells that have entered a stage of exhaustion but are also upregulated on regular T cells that recognize antigen presented by professional APC such as DCs (12). There is a wealth of data demonstrating that PD-1-mediated T cell inhibition occurs during DC–T cell interaction and that disrupting this pathway with antibodies results in enhanced responses of T cells stimulated by DCs (24–27).

Cocultures of T cells with allogeneic monocyte-derived DCs are a widely used model to study T cell responses. In this study, we have exploited this system to assess immune checkpoint inhibitors targeting TIM-3, BTLA, CD160, LAG-3, CTLA-4, and TIGIT alone or in combination with a PD-1 antibody regarding their capacity to enhance T cell proliferation and cytokine production. Moreover, we have analyzed the expression and regulation of these receptors and their ligands on T cells and DCs, respectively. Finally, we have investigated whether differential effects of immune checkpoint inhibitors can be attributed to the T cells or DCs of individual donors. The results of our study highlight the capacity of PD-1 antibodies to enhance CD4 and CD8 T cell responses and, moreover, indicate that antibodies targeting BTLA or TIM-3 might be effective when used in combination with PD-1 antagonists.

## Materials and Methods

### Sample Collection and Cell Isolation

Peripheral blood mononuclear cells (PBMCs) were isolated from heparinized whole blood of healthy volunteer donors (red-cross Austria) by standard density-gradient centrifugation with Lymphoprep (07851, Axis-Shield PoC AS). Donors gave their written informed consent, and approval was obtained from the ethics committee of the Medical University of Vienna (ECS1183/2016). Monocytes were purified using MagniSort CD14 Separation Kits (8802-6834-74, eBioscience). Bulk T cells were purified using MACS Pan T Cell Isolation Kits (130-096-535, Miltenyi). Populations showed at least 95% purity. Cells were either immediately processed or cryopreserved in RPMI medium containing 10% FBS and 10% DMSO for later use. For the generation of immature and mature DCs, monocytes were cocultured with IL-4 (0.1 U/μl) and GM-CSF (50 ng/ml) for 5–6 days, as described previously (28). Mature DCs were generated by the addition of LPS (0.3 μg/ml) as a maturation stimulus for an additional 24 h. Melanoma patient samples were obtained from melanoma patients in regular care at the dermato-oncology out-patient clinic of the medical university of Vienna. The study was approved by the local ethics committee (1210/2012), and informed consent was obtained from the patients.

### Coculture of T Cells and Allogeneic DCs

For T cell proliferation assays, 1–2 × 10^7^ T cells were labeled with 1 μl of a 1 mM CFSE stock solution (C34554, Molecular Probes) in 1 ml PBS for 4 min at room temperature. Subsequently, cells were washed twice with RPMI containing 10% FBS.

CFSE-labeled T cells (1 × 10^5^/well; 1 × 10^6^/ml) were then cocultured with 1.5 × 10^3^ or 6 × 10^3^/well monocyte-derived allogeneic DCs in 96-well round-bottom plates for 6 days, unless indicated otherwise. RPMI-1640 (R8758, Gibco) supplemented with 10% FBS, Penstrep, and Amphotericin was used as a standard cell culture medium.

The following monoclonal-blocking antibodies or combinations thereof were added at a final concentration of 10 μg/ml: TIGIT (clone MBSA43, eBioscience), CTLA-4 (Ipilimumab, Yervoy^®^), Rat IgG_2A_ isotype, and TIM-3 (clones 54447 and 344823 from R&D Systems). Ligand-blocking antibodies to PD-1 (clone MK-3475), BTLA (clone E4H9), CD160 (clone CL1-R2), LAG-3 (clone 25F7), and an isotype control antibody [all human IgG1 carrying two mutations, L234A and L235A, in the CH2 domain of the heavy chain eliminating ADCC and CDC effector functions, as described elsewhere (29)] were produced from publicly available sequences by transient expression in CHO cells and purified using Mabselect SuRe-based affinity chromatography (GE Healthcare). The TIGIT mAb clone MBSA43 was shown previously to enhance HIV-1-specific T cell responses by blocking TIGIT inhibition (30). The TIM-3 antibody clone 344823 was previously used to block TIM-3 (31). The CD160 antibody CL1-R2 was described previously to block interaction with HVEM and enhance HIV-1-specific CD8 T cell responses (32, 33). BTLA Ab E4H9 and LAG-3 Ab 25F7 were validated in Jurkat-based reporter assays, as described in Figure S1 in Supplementary Material.

Culture supernatants were harvested after 6 days of coculture and stored at −20°C, followed by Luminex multiplex cytokine analysis (System 100, Luminex Inc.). The concentration of IFN-γ, IL-2, IL-10, IL-13, IL-17, and TNF-α was measured according to the manufacturer’s instructions.

### Flow Cytometry

Multicolor flow cytometry analysis was performed to assess proliferation and surface-marker expression of resting and activated T cells and DCs using the following mAbs: CD1a-PE (HI149), CD3-PE-Cy7 and BV421 (UCHT1), CD4-PerCP-Cy5.5 (RPA-T4), CD8-BV421 (RPA-T8), CD28-PE (CD28.2), CD66a-PE (ASL-32), CD80-PE (2D10), CD83-PE (HB15e), CD155-PE (SKII.4), CD160-PE and -APC (BY55), CD270-PE (122), CD272-APC (MIH26), CD273-PE (24F.10C12), CD274-PE (29E.2A3), CD279-APC (EH12.2H7), mouse IgG_1_ isotype control-PE and -APC (MOPC-21), and mouse CD45.2-PE-Cy7 (104), all from Biolegend. CD1a-APC and -BV421 (HI149), CD4-PE (RPA-T4), CD8-APC and PE-Cy7 (RPA-T8), CD14-APC (M5E2), and CD25-PECy7 (M-A251) were purchased from BD Pharmingen. CD86-PE (IT2.2), CD152-eF660 (14D3), CD223-PE (3DS223H), CD258-PE (7-3), and TIGIT-PE (MBSA43) antibodies were from eBioscience, and CD366-PE (344823) from R&D Systems. Cells were stained in ice cold FACS buffer (PBS, 1% BSA, and 0.1% NaN_3_) for 30 min. DCs were additionally incubated in 1 mg/ml Beriglobin (300666, CSL Behring) for 20 min on ice before staining to prevent non-specific binding of the mAbs to Fc receptors. 7-AAD (Biolegend) was used to exclude dead cells from the analysis.

Data were acquired on an LSR Fortessa (BD Biosciences) and analyzed using FlowJo 10.2 (Tree Star, Ashland, OR, USA). Fluorescence intensity is shown on a standard logarithmic or biexponential scale.

### Cytotoxicity Assay

Effector T cells from allo-MLRs were harvested on day 6, counted, and incubated with target cells at a ratio of 5:1 or 1:1 for 2–3 h at 37°C before analysis by flow cytometry. Stimulator cells (mouse Bw5147 cells expressing membrane-bound aCD3, abbreviated Bw_aCD3_) were used as target cells, as described previously (34). To determine the specific cell lysis, an equal number of wild-type Bw (Bw_wt_) cells, which lack the expression of aCD3, was added. Bw_wt_ and Bw_aCD3_ were discriminated by staining for CD14, contained in the stem of the aCD3 construct. Percentage of specific lysis was calculated using the following formula:

$$100-\frac{{\text{number of live BW}}_{\text{aCD3}}*100}{{\text{number of live BW}}_{\text{wt}}}.$$

### Data Normalization and Statistical Analysis

Data were normalized to an arbitrary scale using an adaptation of the standard score, calculated with the formula

$$x_{\text{norm}}=\frac{x-\text{baseline}}{\text{SD}},$$

where baseline means the median of the isotype control samples, and SD stands for the standard deviation of all the antibody conditions of a T cell donor. The normalization score shows the number of SDs by which the data point differs from the respective isotype control replicates.

All statistical analyses were performed using Graphpad Prism 7. Proliferation and cytokine data were analyzed with non-parametric repeated measurement ANOVAs (Friedman’s test). Dunn’s multiple comparison *post hoc* test was used to determine differences to the IgG1 isotype or PD-1 control condition. *P* values under 0.05 were considered significant (*), *P* < 0.01 (**), *P* < 0.001 (***), and *P* < 0.0001 (****).

## Results

### Distinct Checkpoint Inhibitor Combinations Boost T Cell Proliferation

**Figure 1:**
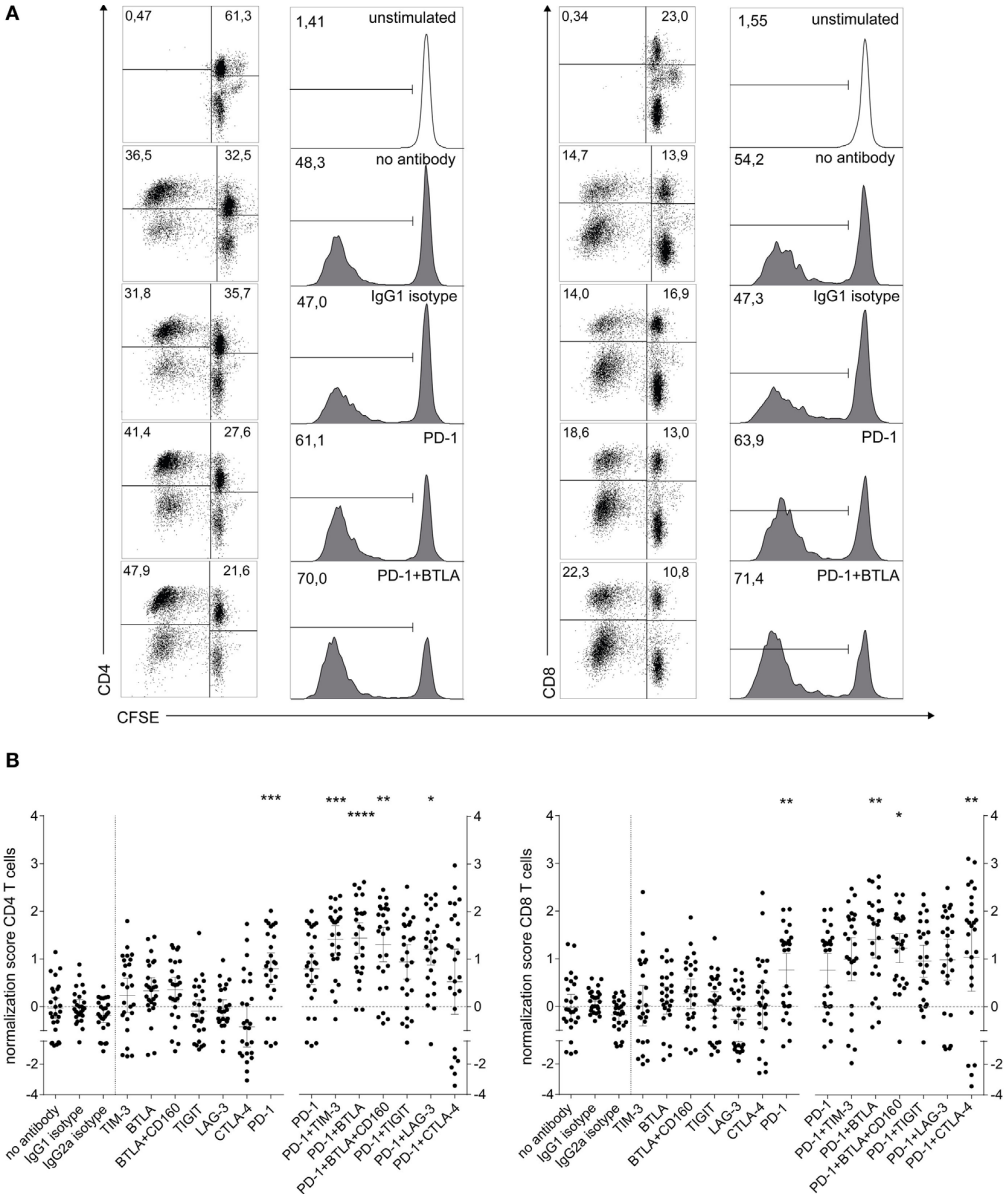
**Influence of checkpoint inhibitors on T cell proliferation during the stimulation of human T cells with allogeneic mature dendritic cells (DCs)**. 1 × 10^5^ CFSE-labeled human T cells were stimulated with 6 × 10^3^ mature allogeneic human monocyte-derived DCs in the presence of blocking antibodies to the indicated molecules. After 6 days of coculture, the cells were stained for CD4 and CD8 and analyzed by flow cytometry. 7AAD^+^ cells were excluded from the analysis. **(A)** T cell proliferation during allo-MLR of a representative T cell donor, showing dot plots and histograms of isotype, PD-1 antibody, and PD-1 + BTLA antibody conditions for CD4 and CD8 T cells. Dot plots show CFSE versus CD4 (left panel) or CD8 (right panel) in live cells. Histograms show the percentage of CFSE^low^ cells gated on live CD4 or CD8 T cells, respectively. **(B)** Normalized proliferation scores (as described in Section “Materials and Methods”) of CD4 T cells (left panel) and CD8 T cells (right panel) of 26 healthy T cell donors are shown. Each data point represents the mean of triplicates of one T cell donor (mean ± 95% CI). Stars indicate significant differences compared to IgG1 isotype control (single antibody conditions) or PD-1 antibody (PD-1 antibody containing conditions). The PD-1 dataset was included in both analyses. All *P* values were calculated using Dunn’s multiple comparison *post hoc* test following a Friedman ANOVA (*P < 0.05, **P < 0.01, and ***P < 0.001).

We used cocultures of human CFSE-labeled T cells with allogeneic DCs to evaluate the capacity of immune checkpoint inhibitors targeting TIM-3, BTLA, CD160, TIGIT, LAG-3, and CTLA-4 alone or in combination with a PD-1 blocker to enhance human T cell responses. We selected antibody clones that were previously described to potently block interaction with their ligands, and in addition validated the BTLA and LAG-3 antibodies using a Jurkat-based reporter platform (as described in Section “Materials and Methods” and Figure S1 in Supplementary Material). Stimulation of T cells derived from a representative donor with (6 × 10^3^) mature DCs resulted in high percentages of CFSE^low^ T cells in the CD4 and CD8 subsets. Addition of a PD-1-blocking antibody enhanced T cell proliferation in both subsets, and a combination of immune checkpoint inhibitors targeting PD-1 and BTLA further augmented CFSE^low^ CD4 and CD8 T cells (Figure 1A). Analysis of data from 26 healthy donors underlines the potency of PD-1 to enhance CD4 and CD8 T cell proliferation (Figure 1B). Although a tendency of TIM-3 and BTLA antibodies to increase CD4 T cell proliferation was observed, blocking antibodies targeting co-inhibitors other than PD-1 generally failed to significantly increase T cell proliferation when used alone. By contrast, antibodies to several immune checkpoints had the capacity to further increase T cell proliferation when combined with PD-1 blockade. Antibodies to TIM-3 and LAG-3 augmented CD4 T cell proliferation, and the CTLA-4 antibody ipilimumab increased the proliferation in the CD8 subset when used in combination with PD-1 blockade. Importantly, the BTLA antibody was the only agent able to significantly enhance PD-1 effects in both CD4 and CD8 T cells. The addition of a blocking antibody to CD160, which shares the ligand HVEM with BTLA, did not further enhance the effect of BTLA blockade (Figure 1B). Although T cell proliferation in response to allogeneic stimulation depended on the number and maturation status of DCs, comparable effects of immune checkpoint inhibitors were also observed in cocultures with immature DCs or lower numbers of DCs (Figure S2 in Supplementary Material). Importantly, when using PBMC from patients with melanoma in allogeneic coculture assays, we could observe a similar tendency of the PD-1/TIM-3 and PD-1/BTLA combinations to enhance proliferation, with TIM-3 being particularly effective in both CD4 and CD8 T cells (Figure S3 in Supplementary Material).

### PD-1 Blockade Has a Unique Impact on Cytokine Production

**Figure 2:**
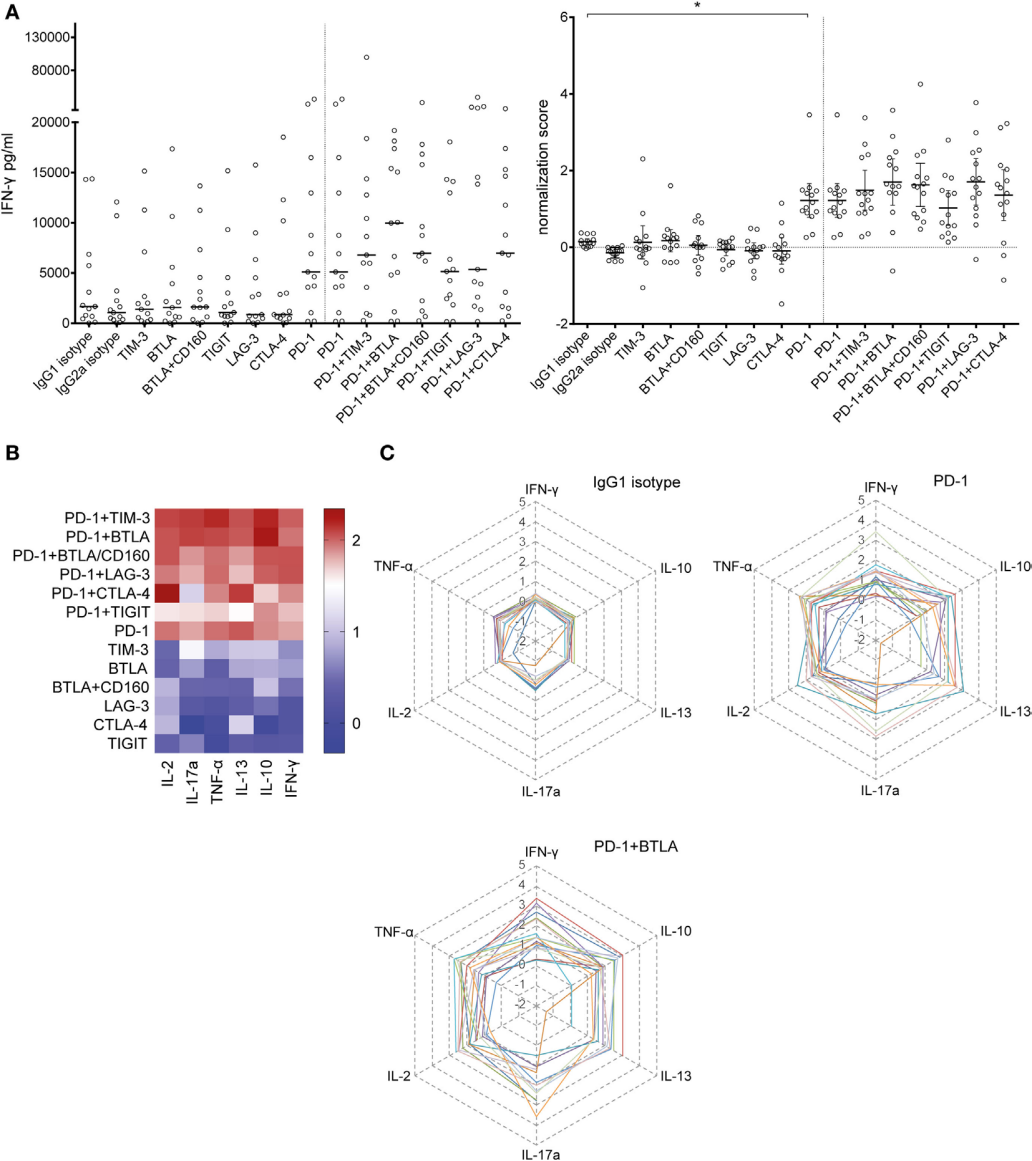
**Effect of blocking antibodies to coinhibitory molecules on the cytokine content in the culture supernatants**. Culture supernatants of T cell proliferation assays were collected and analyzed using a Luminex™-based multiplexing assay. Data from 14 T cell donors cocultured with 6 × 10^3^ mature allogeneic dendritic cells are shown. **(A)** The IFN-γ content in the culture supernatant increases with the addition of a PD-1-blocking antibody. The median of the absolute amount of IFN-γ (left panel) and the deduced normalization values (mean ± 95% CI, number of SDs above the median of the isotype control replicates) are shown (right panel). **(B)** Summary of the cytokines simultaneously measured in the culture supernatants, depicted as a heat map. The mean normalization values of 14 donors are shown for IL-2, IL-17a, TNF-α, IL-13, IL-10, and IFN-γ. **(C)** Radar plots showing the IL-2, IL-17a, TNF-α, IL-13, IL-10, and IFN-γ content for the IgG1 isotype control, PD-1, and PD-1 + BTLA antibody conditions. Each line represents normalized data of one individual donor (*n* = 14).

In order to locate possible skews in the cytokine pattern, we analyzed the concentration of multiple cytokines in the culture supernatants of T cell proliferation assays using Luminex™-based multiplexing. Our results show that PD-1 blockade greatly increases the IFN-γ content in the culture supernatant (Figure 2A). Analysis of IL-2, IL-10, IL-13, IL-17a, and TNF-α pointed to a unique potency of PD-1 blockade to enhance cytokine production in T cell stimulation cultures (Figures 2B,C; Figure S4 in Supplementary Material). PD-1 in combination with TIGIT blockade reduced IL-2 and IL-13 levels compared to PD-1 blockade alone (Figure S4 in Supplementary Material). Blocking BTLA alone or together with PD-1 had the tendency to increase cytokine production but the effect did not reach statistical significance (Figure 2; Figure S4 in Supplementary Material).

### Expression of Coinhibitory Receptors on T Cells Stimulated by Allogeneic DCs

**Figure 3:**
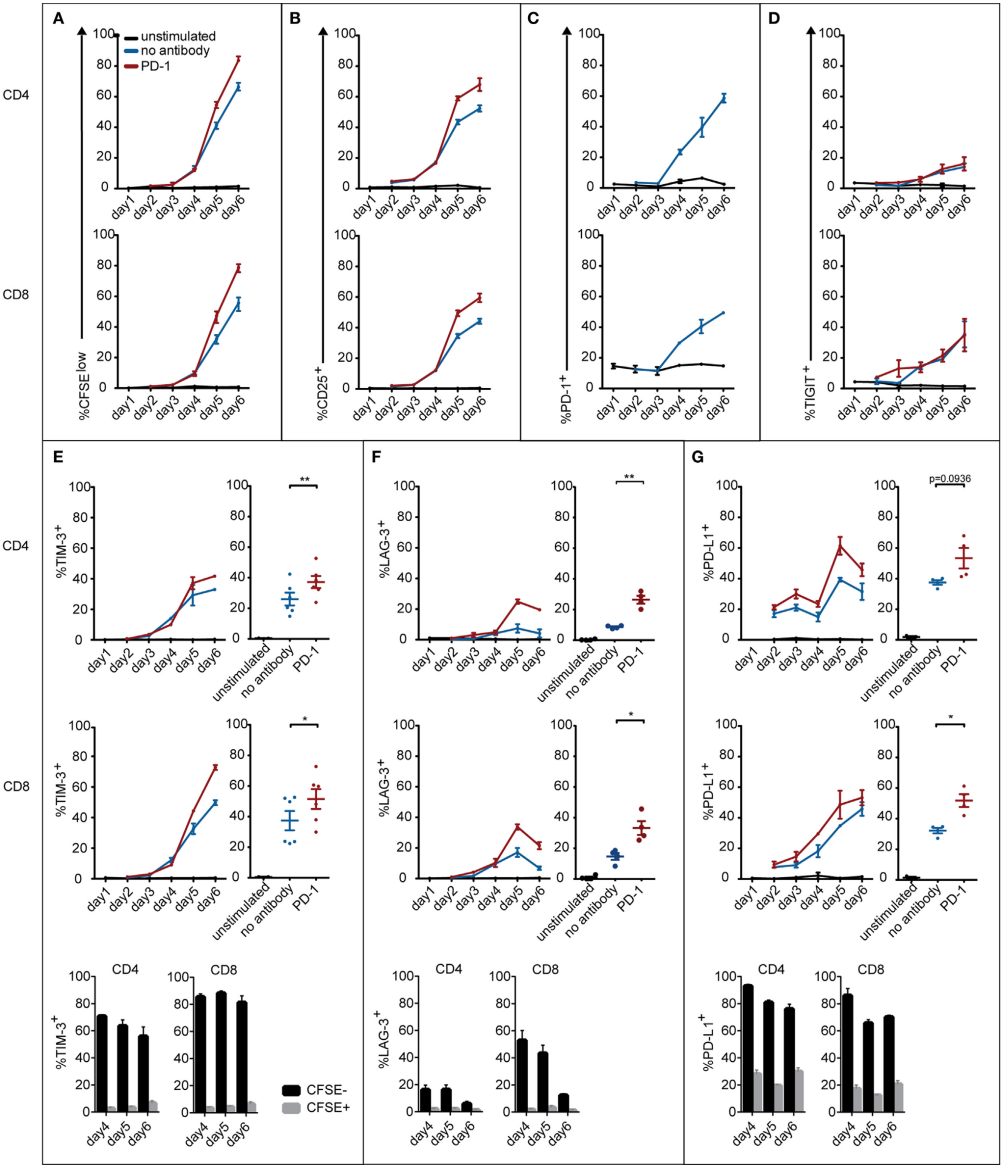
**Expression of coinhibitory molecules on CD4 and CD8 T cells during the course of an MLR**. T cells were left unstimulated or cocultured with mature allogeneic dendritic cells for 2–6 days in presence or absence of a PD-1-blocking antibody. At the indicated time points, CD4 and CD8 T cells were analyzed for CFSE dilution **(A)**, expression of the activation marker CD25 **(B)**, and the coinhibitory molecules PD-1 **(C)**, TIGIT **(D)**, TIM-3 **(E)**, LAG-3 **(F)**, and PD-L1 **(G)**. Line graphs represent mean ± SEM of technical triplicates from one representative donor. Panels **(E–G)** additionally show cumulative data from day 6 of three to six donors and the percentage of CD4 and CD8 T cells expressing the indicated marker in the CFSE^high^ and CFSE^low^ subset.

It is evident that the efficacy of blocking antibodies critically depends on the expression of their antigens on T cells. Since it is known that immune checkpoints like PD-1, TIGIT, TIM-3, LAG-3, and also PD-L1 are strongly upregulated during T cell activation, we set out to monitor the expression of these markers in T cells stimulated with allogeneic DCs. As expected we observed a strong increase of CFSE^low^ and CD25^+^ T cells in the CD4 and CD8 subsets over the 6-day time course of the experiment (Figures 3A,B). PD-1 expression was absent in unstimulated CD4 T cells and low in unstimulated CD8 T cells. T cells strongly upregulated PD-1 when cultured in presence of allogeneic DCs, but not when cultured alone (Figure 3C). TIGIT expression on CD4 and CD8 T cells also increased with the duration of stimulation, but was not influenced by PD-1 blockade (Figure 3D). TIM-3, LAG-3, and PD-L1 were upregulated in both CD4 and CD8 T cells upon stimulation. Importantly, PD-1 blockade further increased the percentage of T cells positive for these molecules (Figures 3E–G). Furthermore, we found that PD-1, TIGIT, TIM-3, LAG-3, and PD-L1 expression was selectively upregulated in T cells that responded to allogeneic DCs, since these markers were mainly confined to T cells that had divided and expressed the activation marker CD25 (Figures 3E–G; data not shown). In contrast to the other immune checkpoints analyzed in our study, we observed that BTLA was expressed in the majority of freshly isolated T cells, which is in line with previous analyses (35). We observed a trend of BTLA downregulation in T cell cocultures with allogeneic DCs (data not shown).

### T Cell Cytotoxic Capacity in Response to Checkpoint Inhibition

**Figure 4:**
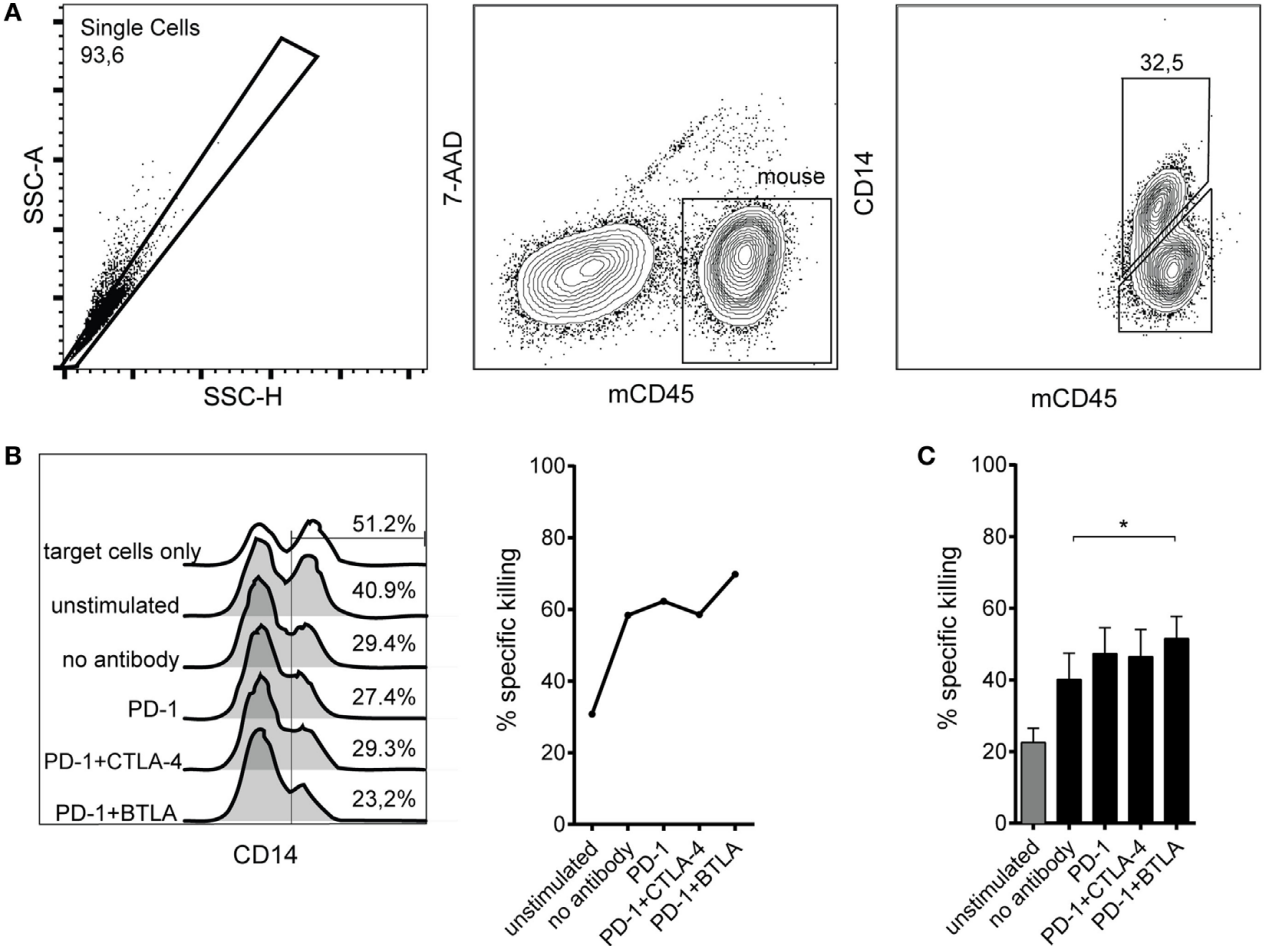
**Cytotoxic capacity of T cells stimulated by allogeneic dendritic cells (DCs) in presence of checkpoint inhibitors**. T cells were stimulated with allogeneic DCs for 6 days without antibodies or in presence of blocking antibodies to the indicated coinhibitory receptors. Subsequently, their capacity to kill target cells harboring a membrane-bound anti-CD3 antibody fragment was assessed by incubating them with a 1:1 mixture of Bw_wt_ and Bw_aCD3_ target cells. The relative loss of CD14^+^ Bw_aCD3_ target cells in the coculture was analyzed by flow cytometry and used to calculate the percentage of specific killing. **(A)** Representative FACS plots from one donor showing allo-stimulated T cells in coculture with Bw cells. The murine Bw cells were detected using an antibody to murine CD45, and a CD14 antibody was used to detect the target cells *via* the CD14 stem of the anti-CD3 antibody fragment. **(B)** Representative data from one donor showing the abundance of CD14^+^ Bw_aCD3_ target cells and the calculated percentage of specific killing. **(C)** Cumulative data from six donors of three independent experiments are shown. Data represent mean ± SEM.

The ultimate goal of immune checkpoint inhibitor-based therapies is to promote the eradication of tumors by effector cells. We chose to analyze PD-1 antibodies and immune checkpoint combinations that showed efficacy in enhancing CD8 T cell proliferation regarding their ability to promote cytotoxic effector function. T cells stimulated by allogeneic DCs for 6 days were harvested, counted, and cocultured with murine Bw5147 cells expressing a membrane-bound anti-CD3 antibody, which served as target cells for TCR complex-mediated killing as described before (34). Bw5147 cells lacking anti-CD3 expression were used to control for unspecific cytotoxic activity (Figure 4A). While T cells stimulated by allogeneic DCs in presence of PD-1 antibodies showed a trend to increase the percentage of specific killing, only the combined use of PD-1 and BTLA antibodies resulted in significantly increased killing compared to allo-stimulated T cells without the addition of a blocking antibody (Figures 4B,C).

### Upregulation of Costimulatory Molecules on DCs in Coculture

**Figure 5:**
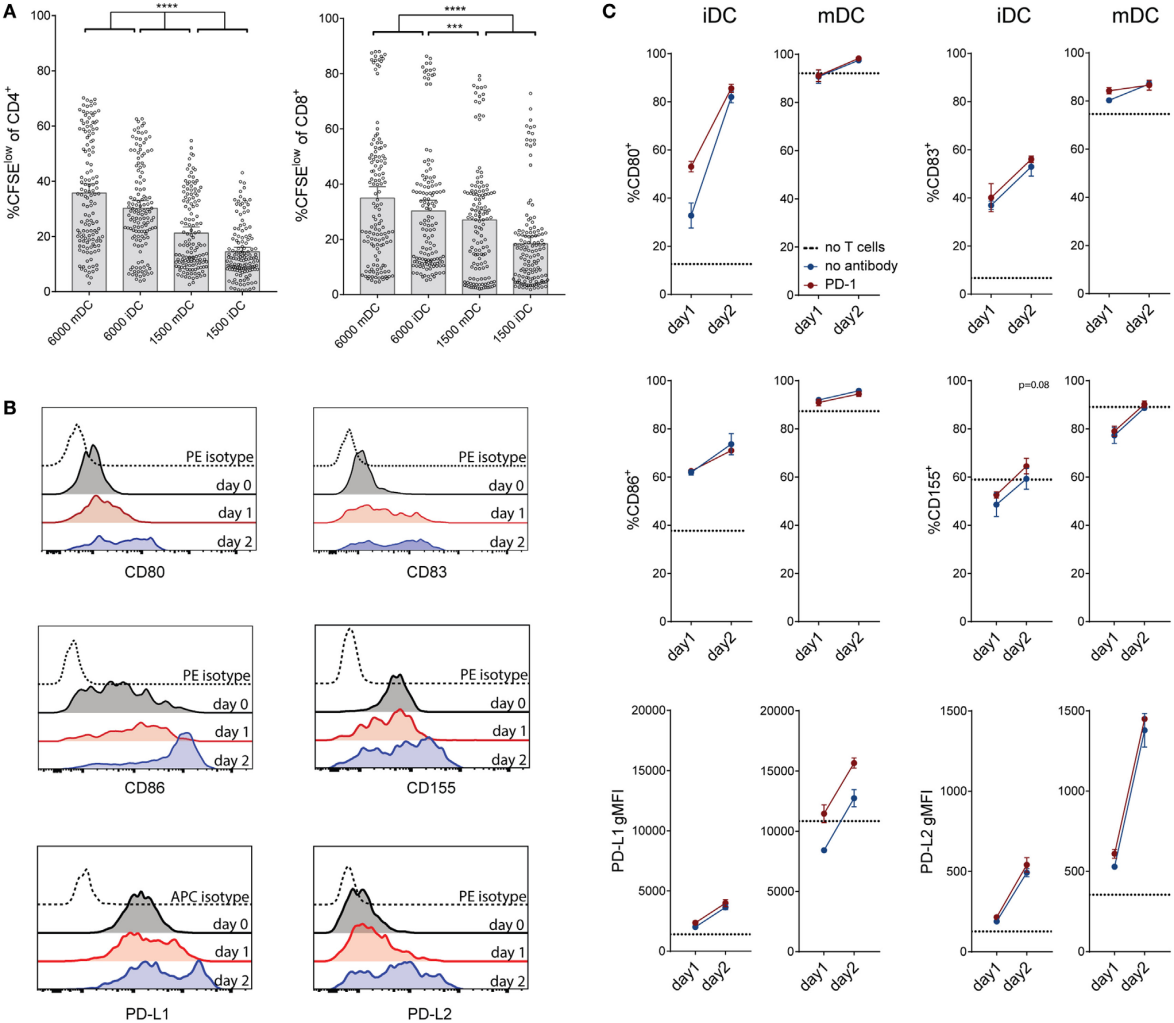
**Effect of dendritic cell (DC) number and maturation status on T cell proliferation, and changes of DC marker expression during coculture**. **(A)** Comparison of the effect of different amounts of immature and mature DCs on T cell proliferation. Data are expressed as %CFSE^low^ cells of live CD4^+^ or CD8^+^ T cells. Each dot represents data from one antibody condition of one of 10 T cell donors. Mean with 95% CI is shown. Significances were calculated using RM one-way ANOVA with Tukey’s multiple comparisons *post hoc* test. **(B)** Immature DC expression of the costimulatory markers CD80, CD83, and CD86 and the coinhibitory markers CD155, PD-L1, and PD-L2 before coculture with allogeneic T cells, and after 24 or 48 h of coculture. Data from one donor, representative of at least three different experiments, are shown. **(C)** Measurement of costimulatory markers on immature and mature DCs after 24–48 h of coculture with allogeneic T cells, with (red lines) or without (blue lines) the addition of a PD-1-blocking antibody. Dotted lines represent the expression of the respective marker on DCs before coculture with T cells. Data from one donor (mean ± SEM of triplicates) are shown.

It is well established that the maturation status of DCs has critical impact on their T cell stimulatory capacity. Upon LPS treatment, immature DCs strongly upregulated the maturation marker CD83 and also costimulatory ligands and MHC expression, which are crucial for their ability to induce vigorous T cell responses (Figure S5 in Supplementary Material; data not shown). Compared to immature DCs, the LPS-treated DCs induced increased proliferative responses in CD4 and in CD8 T cells (Figure 5A). We observed that immature DCs strongly upregulated the costimulatory molecules CD80, CD83, and CD86 and the coinhibitory markers CD155, PD-L1, and PD-L2 within 24–48 h of coculture (Figure 5B). As shown in Figure 5C, mature DCs also changed their expression profile during coculture, further upregulating PD-L1 and PD-L2. Interestingly, the presence of a PD-1-blocking antibody seemed to accelerate maturation, i.e., CD80 and CD83 expression, of iDCs. Furthermore, CD155 and PD-L1 expression was increased in presence of PD-1-blocking antibodies (Figure 5C).

### Contribution of T Cells and DCs to Effects of Checkpoint Inhibition

**Figure 6:**
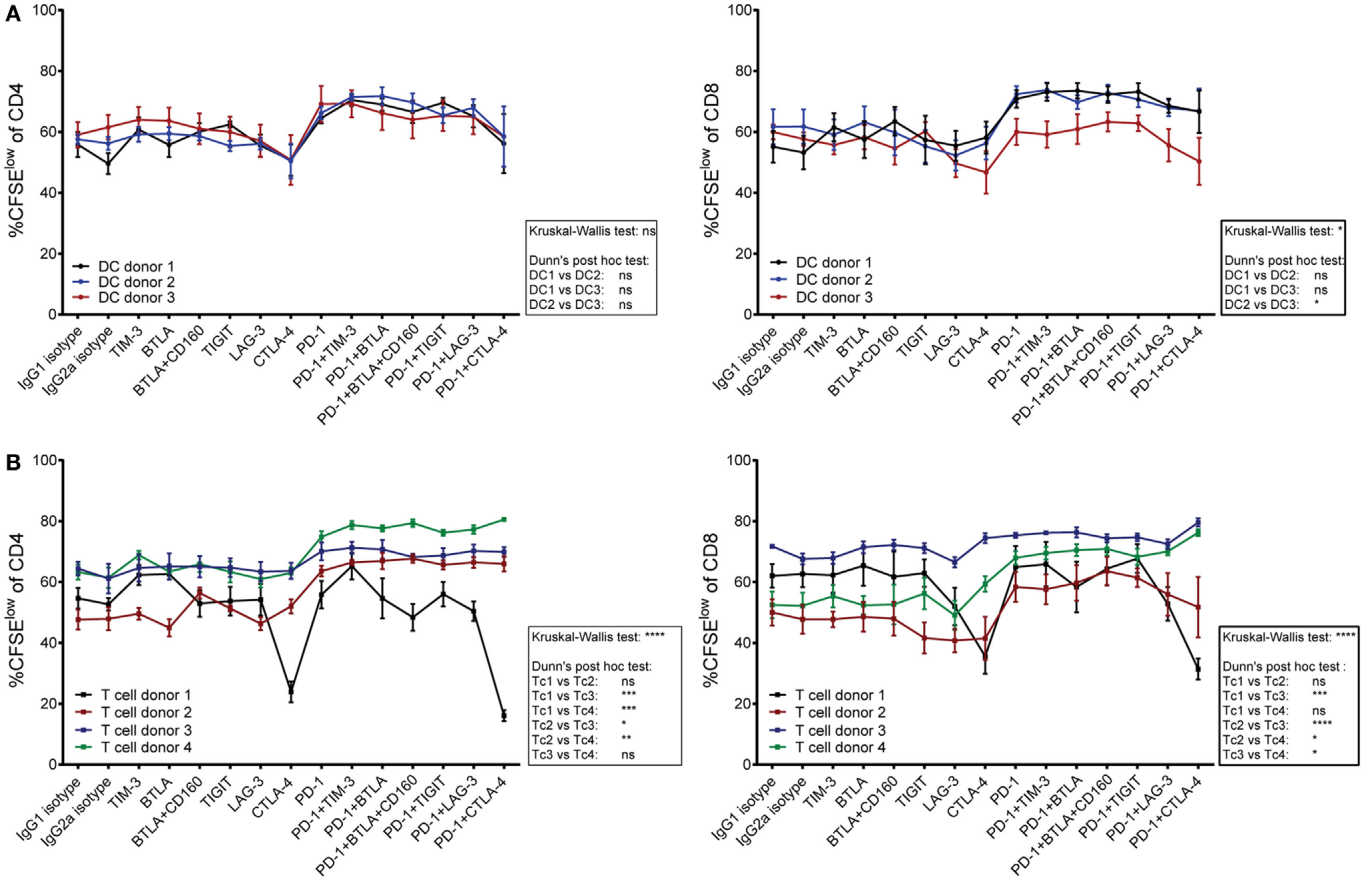
**The contribution of T cells versus dendritic cells (DCs) on the effect of checkpoint inhibitors on T cell proliferation**. T cells derived from four donors were stimulated for 6 days with mature DCs from three allogeneic donors in the presence of blocking antibodies to the indicated molecules. The data were both analyzed by plotting proliferation data from different T cell donors that were stimulated by an individual DC donor, and *vice versa*. **(A)** The data points in each line represent proliferation of T cells derived from four different donors upon stimulation with DCs derived from one individual donor. **(B)** The data points in each line represent proliferation of T cells derived from one individual donor in response to stimulation with DCs from three different donors. **(A,B)** The percentages of CFSE^low^ CD4 T cells (left panels) and CFSE^low^ CD8 T cells (right panels) are shown. Data represent mean ± SEM. Differences between donors were calculated by a Dunn’s *post hoc* test following a Kruskal–Wallis test.

Donor compatibility of T cells and allogeneic DCs, as well as coinhibitory ligand availability on DCs, might influence T cell proliferation in response to checkpoint inhibition. Since the effects of immune checkpoint inhibitors differed between cocultures, we performed a set of experiments to address whether these differential effects can be attributed to the DCs or the T cells in the cocultures. T cells derived from four different donors were each stimulated with DCs derived from three unrelated donors. The results were displayed by combining data from four different T cell donors stimulated with DCs from one individual donor (Figure 6A) and by combining data of individual T cells donors stimulated by DCs derived from three different donors (Figure 6B). The results indicate that T cells derived from different donors varied considerably regarding their response to different checkpoint inhibitors, whereas the choice of DC donor appeared to have minor impact.

In summary, we could show that single checkpoint inhibitors, except PD-1, do not greatly increase proliferation or change the cytokine pattern in healthy donors. In combination with PD-1, however, multiple checkpoint inhibitors were able to augment CD4 and CD8 T cell proliferation compared to PD-1 alone. We found that many of these checkpoint molecules are upregulated on T cells upon PD-1 blockade, and coinhibitory as well as costimulatory molecules are also enhanced on allo-DCs at the beginning of coculture and in response to PD-1 blockade.

## Discussion

Despite the great interest in the use of immune checkpoint inhibitors and combinations thereof to enhance T cell responses in cancer patients, there are surprisingly few studies that have compared different immune checkpoint inhibitors in parallel. Here, we provide a comparison of immune checkpoint inhibitors targeting several different T cell coinhibitory receptors regarding their capacity to enhance human T cell responses when used alone or in combination with a PD-1 blocker. Cocultures of T cells with allogeneic monocyte-derived DCs, as used in our study, are a widely used model system to assess human T cell responses *in vitro*. DCs express high levels of coinhibitory ligands including CD80 and CD86, PD-L1 and -2, MHC class II and CD155. DCs are thought to be required for the efficient activation of naïve T cells. Thus, this model system integrates all elements of DC–T-cell interaction including most costimulatory and coinhibitory pathways and avoids a restriction bias to certain antigens. Certain DC subsets are endowed with the capacity to efficiently cross-present antigens, suggesting an essential role of DCs in immune responses toward tumors (23, 36). Therefore, the ability to enhance DC-mediated T cell activation will possibly be predictive for the therapeutic efficacy of immune checkpoint inhibitors.

We observed that none of the tested antibodies were efficient in significantly enhancing T cell responses when used alone, except for the PD-1 antibody, which strongly enhanced proliferation of both CD4 and CD8 T cells and cytokine production. Even the CTLA-4 inhibitor ipilimumab that has successfully been used in the clinic for treatment of metastatic melanoma did not enhance proliferation in our setting when used as a single agent. In this context, it should be noted that recent work suggests that the therapeutic efficacy of CTLA-4 antibodies may not be primarily due to neutralization of CTLA-4-mediated T cell inhibition but is owed to its ability to deplete regulatory T cells in the tumor microenvironment (37, 38). Importantly, we could observe that blockade of TIM-3, BTLA, LAG-3, and CTLA-4 in combination with PD-1 increased proliferation of at least one T cell subset compared to PD-1 alone. This is in line with previous studies that reported synergistic effects between PD-1 and TIM-3 (39), BTLA (40), LAG-3 (41), and CTLA-4-blocking antibodies (11, 42). By contrast, a TIGIT-blocking antibody did not enhance T cell responses when used alone or in combination with PD-1 blockade. We observed that TIGIT is only weakly expressed in T cells cocultured with allogeneic DCs, which could be a potential reason for the failure of TIGIT blockade to enhance T cell responses in our study. BTLA together with PD-1 antibody was the only combination tested that enhanced both CD4 and CD8 T cell proliferation compared to PD-1 antibody alone. Moreover, we provide preliminary data indicating that T cells stimulated in presence of blocking antibodies to BTLA and PD-1 acquire potent cytotoxic capacity. Derré et al. previously demonstrated persistent BTLA expression and HVEM-mediated inhibition of human tumor-specific CD8 T cells (18). Thus, blockade of BTLA might have potential in cancer therapy. However, the pathways involving BTLA are complex, since HVEM, which is the sole binding partner for BTLA known to date, also functions as a costimulatory receptor. In fact, BTLA itself has been shown to also act as an activating ligand for HVEM that promotes cell activation and survival (43). Thus, BTLA blockade likely disrupts not only coinhibitory BTLA signaling but also activating signaling *via* the TNFR-SF-member HVEM. Additionally, if other HVEM ligands like CD160 or LIGHT are present, HVEM costimulation may be maintained upon BTLA blockade. We have observed that coblockade of the putative coinhibitor CD160 did not enhance T cell responses, which could be explained by a minor significance of CD160 inhibition in this setting, but also by reduced costimulation *via* HVEM under conditions where two of its ligands are blocked. Given the complexity of BTLA pathways, further studies are required to assess the potential of BTLA blockers to release T cell inhibition without reducing T cell responses *via* HVEM.

Moreover, our results indicate that PD-1 blockade strongly augments production of cytokines in the cocultures. In line with the proliferation data, we observed only minor changes in the cytokine content of the culture supernatants in response to blockade of other immune checkpoints. Our data indicate that immune checkpoint inhibitors do not polarize T cells into a specific direction in this system.

Monitoring marker expression in T cell–DC stimulation cultures, we could observe profound changes in both cell types. Immature DCs had a reduced ability to induce T cell proliferation compared to mature DCs, but they quickly upregulated costimulatory and coinhibitory molecules in coculture with T cells, acquiring a phenotype that resembles mature DCs. The contact with allogeneic T cells, ligation of CD40, and the inflammatory cytokine environment are a likely maturation stimulus: PD-L1 expression, for example, is known to be upregulated on APC *in vitro* in response to IFN-γ (44) and the common γ chain cytokines IL-2, IL-7, and IL-15 (45). Additional interactions might contribute to DC maturation, e.g., the engagement of MHC class II molecules by LAG-3, which is upregulated on activated T cells (46).

As expected, we could observe a strong upregulation of coinhibitory molecules, such as PD-1, TIM-3, and LAG-3, and also PD-L1 in T cells that were stimulated by allogeneic DCs. Importantly, PD-1 blockade enhanced the upregulation of activation markers on both DCs and T cells in the cocultures. Increased expression of potential immune checkpoints like TIM-3 and LAG-3 on T cells upon PD-1 blockade could explain our observation that immune checkpoint inhibitors have enhanced efficacy when used in combination with PD-1 antibodies. BTLA is distinct in its expression pattern, being present in naïve T cells, but downregulated in effector cells (18). In line with our observations, it has previously been reported that BTLA blockade is more effective under strong allo-stimulation conditions (47). The mechanism behind the synergy between BTLA and PD-1 blockade detected in our study, however, remains to be determined.

We observed varying effects of distinct immune checkpoint inhibitors depending on the choice of the allogeneic donors. Consequently, we performed a set of experiments to address whether this can be attributed to the DCs or the T cells in the cocultures. Our results suggest that the T cells rather than the DCs play a decisive role in the outcome of immune checkpoint blockade. Assessment of parameters like immune cell infiltration and immune checkpoint expression might help to personalize immune therapy of cancer in the near future (48). In this context, it would be interesting to explore whether the response of patient T cells to blocking antibodies to coinhibitory pathways *in vitro* is predictive for the outcome of immune checkpoint inhibitor treatment. Importantly, in experiments with a small set of PBMC from patients with melanoma, we observed results comparable to those obtained with T cells from healthy donors regarding the effect of checkpoint inhibitor combinations. Immune checkpoints operate on different levels regulating both T cell activation by DCs and the effector function of antigen-specific T cells (49). Our system might be especially suited to model the effect of immune checkpoint inhibitors during the interaction with DCs, whereas the effect of immune checkpoint inhibitors in the effector phase is difficult to assess *in vitro*.

Our study is the first to compare the effect of different immune checkpoint inhibitors alone or in combination with PD-1 antibodies in human T cell–DC interaction. Our results point to a unique potency of PD-1 blockade in enhancing the T cell response and indicate that harnessing the synergistic effects of blocking coinhibitory pathways might be a promising strategy to increase immune responses. Several immune checkpoint inhibitors were capable to enhance T cell proliferation in presence of PD-1 antibodies and thus might have potential in cancer immunotherapy. Studies in mice have shown that combinations of immune checkpoints with agonistic antibodies to activating T cell costimulatory receptors like 4-1BB potently enhance antitumor responses (50–53). *In vitro* studies such as the one presented here might also prove useful to explore the potential of such combinations in enhancing T cell immunity. The model system employed in our study will help to establish a functional profile of human immune checkpoints and provides the means to screen for the most effective antibody combinations at an early preclinical stage.

## Ethics Statement

This study was carried out in accordance with the recommendations of the ethics committee of the Medical University of Vienna. All subjects gave written informed consent in accordance with the Declaration of Helsinki. The protocol was approved by the ethics committee of the Medical University of Vienna (ECS1183/2016 and ECS1210/2012).

## Author Contributions

PS and CS designed experiments, analyzed data, and wrote the manuscript. CS performed the majority of experiments. CB and JL performed experiments. KG-P and CH provided patient samples and clinical data. MZ and GZ provided essential reagents. All the authors contributed in writing and critically revising the manuscript.

## Conflict of Interest Statement

MZ is an employee of Boehringer Ingelheim. All other authors declare no potential conflict of interest.
