# Supplementary material for: PD-1 Blockade Promotes Emerging Checkpoint Inhibitors in Enhancing T Cell Responses to Allogeneic Dendritic Cells
Source: Front Immunol. 2017 May 22;8:572. doi: 10.3389/fimmu.2017.00572 (PMC5439058; doi:10.3389/fimmu.2017.00572)
Supplement: Supplementary file 1 [file Data_Sheet_1.DOCX]

Supplementary Material

PD-1 blockade promotes emerging checkpoint inhibitors in enhancing T cell responses to allogeneic DC

Carmen Stecher^1^, Claire Battin^1^, Judith Leitner^1^, Markus Zettl^2^, Katharina Grabmeier-Pfistershammer^3^, Christoph Höller^4^, Gerhard J. Zlabinger^3^, and Peter Steinberger^1*^

^1^Division of Immune Receptors and T cell Activation, Institute of Immunology, Center for Pathophysiology, Infectiology and Immunology, Medical University of Vienna, Vienna, Austria.

^2^Cancer Immunology and Immune Modulation, Boehringer Ingelheim RCV GmbH & CoKG, Vienna, Austria

^3^Division of Clinical and Experimental Immunology, Institute of Immunology, Center for Pathophysiology, Infectiology and Immunology, Medical University of Vienna, Vienna, Austria.

^4^Department of Dermatology, Medical University of Vienna, Vienna, Austria

# * Correspondence: Peter Steinberger [peter.steinberger@meduniwien.ac.at](mailto:peter.steinberger@meduniwien.ac.at)

# Supplementary Figures


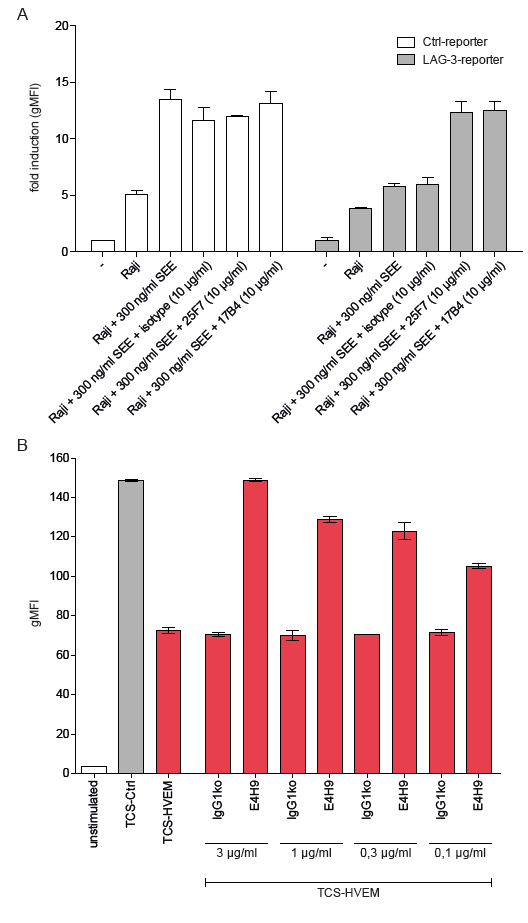


**Supplementary Figure 1.** (previous page) **Validation of monoclonal blocking antibodies 25F7 to LAG-3 and E4H9 to BTLA**. Blocking antibodies to LAG-3 (25F7, Thudium et al., 2011; EP2320940 A2) and BTLA (E4H9, Korman et al., 2013; US8580259 B2) expressed as described in the Material and Methods section were tested in functional assays based on Jurkat NF-κB-eGFP transcriptional reporter cells. **(A)** Jurkat control reporter cells (Ctrl-reporter) and Jurkat reporter cells expressing human LAG-3 were stimulated by the human B-cell line Raji in presence of superantigen (staphylococcal enterotoxin E; SEE) to induce TCR-engagement via the LAG-3 ligand MHC class II. Antibodies to LAG-3 (clones 25F7 and 17B4 (Abcam)) and a human IgG1ko isotype control antibody were added at co-culture onset. Following 24 hours of stimulation, cells were harvested and eGFP expression in the reporter cells was measured by flow cytometry. Reporter activation is shown as fold induction compared to the unstimulated reporter cells. A representative experiment performed in duplicates is shown. **(B)** Jurkat reporter cells expressing BTLA were stimulated with control-T cell stimulators (TCS-Ctrl) and TCS expressing high levels of human HVEM (TCS-HVEM, shown in red). TCS are Bw5147 cells engineered to express membrane-bound anti-CD3 and thus can give Signal 1 to human T cells or human T cell lines upon co-culture (Leitner et al, JIM 2010).


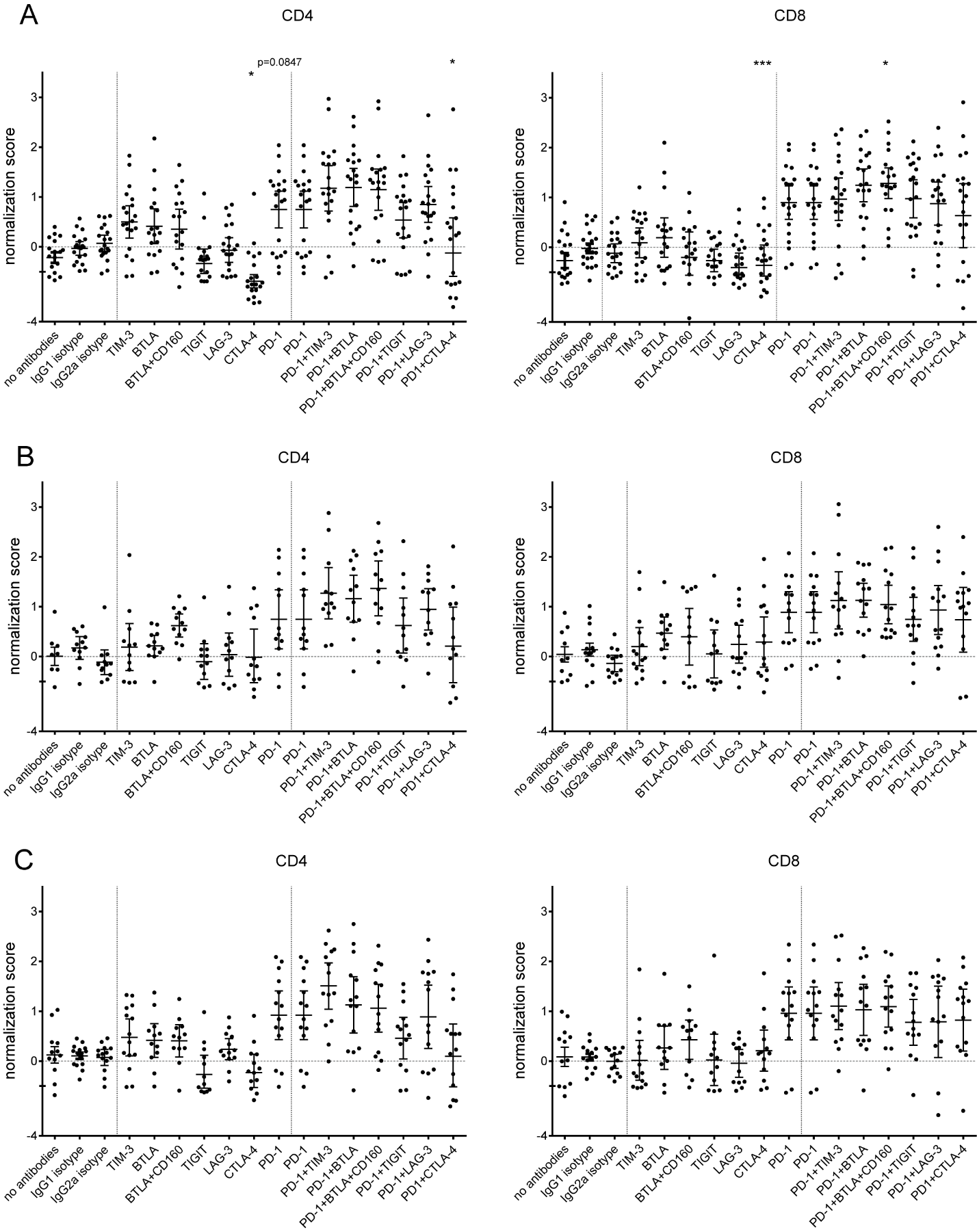
**Supplementary Figure 2.** **Influence of checkpoint inhibitors on T cell proliferation during co-culture of T cells with different amounts of immature or mature allogeneic dendritic cells**. 1x10^5^ CFSE-labeled human T cells were stimulated either with 6x10^3^ immature allogeneic DC **(A)**, 1.5x10^3^ mature **(B)**, or 1.5x10^3^ immature **(C)** allogeneic DC in the presence of blocking antibodies to the indicated molecules. T cells were stained for CD4 and CD8 after 6 days of co-culture and analyzed by flow-cytometry. 7AAD^+^ cells were excluded from the analysis. Normalized proliferation scores (as described in Materials and Methods) of CD4 and CD8 T cells of 20 **(A)** or 14 **(B, C)** healthy T cell donors are shown. Each data-point represents the mean of triplicates of one T cell donor (mean ± 95% CI). Stars indicate significant differences compared to IgG1 isotype control (single antibody conditions) or PD-1 antibody (PD-1 antibody containing conditions), respectively. P values were calculated using Dunn’s multiple comparison post hoc test following a Friedman ANOVA. * P < 0.05, ** P < 0.01, *** P < 0.001.


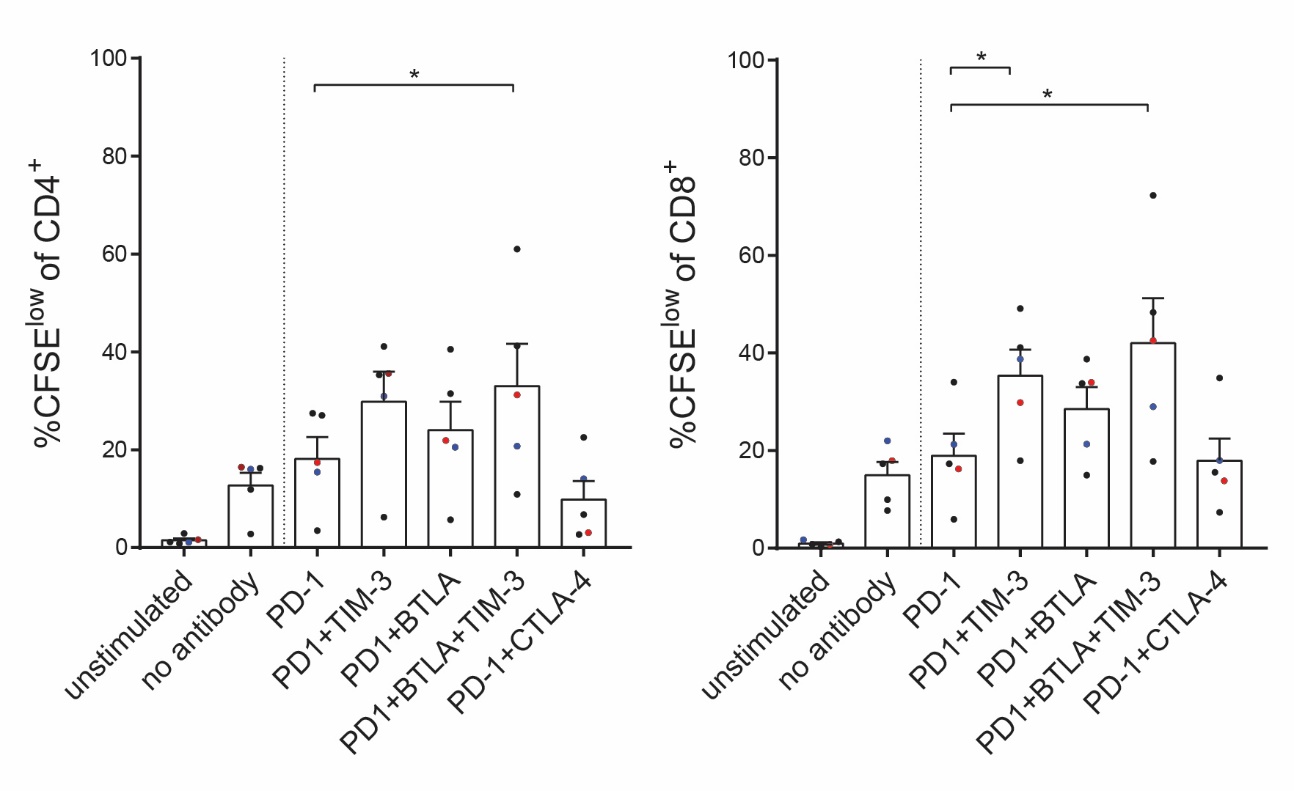

**Supplementary Figure 3.** **Influence of checkpoint inhibitors on T cell proliferation during** c**o-culture of PBMC from melanoma patients with mature allogeneic dendritic cells**. 2x10^5^ CFSE-labeled PBMC from five patients with melanoma were stimulated with 6x10^3^ mature allogeneic DC in the presence of blocking antibodies to the indicated molecules. PBMC were stained for CD3, CD4, CD8 and CD25 after 6 days of co-culture and analyzed by flow-cytometry. 7AAD^+^ cells were excluded from the analysis. The percentage of CFSE^low^ cells in the CD4 (left panel) or CD8 T cell compartment (right panel) of five donors is shown (mean ± SEM). Patients were all male with melanoma stage II or IV and no treatment, or previous rounds of treatment with nivolumab (two patients, highlighted as red or blue dots, respectively). Stars indicate significant differences of the PD-1 combinations to PD-1 antibody alone. P values were calculated using Dunn’s multiple comparison post hoc test following a Friedman ANOVA. * P < 0.05, ** P < 0.01, *** P < 0.001.


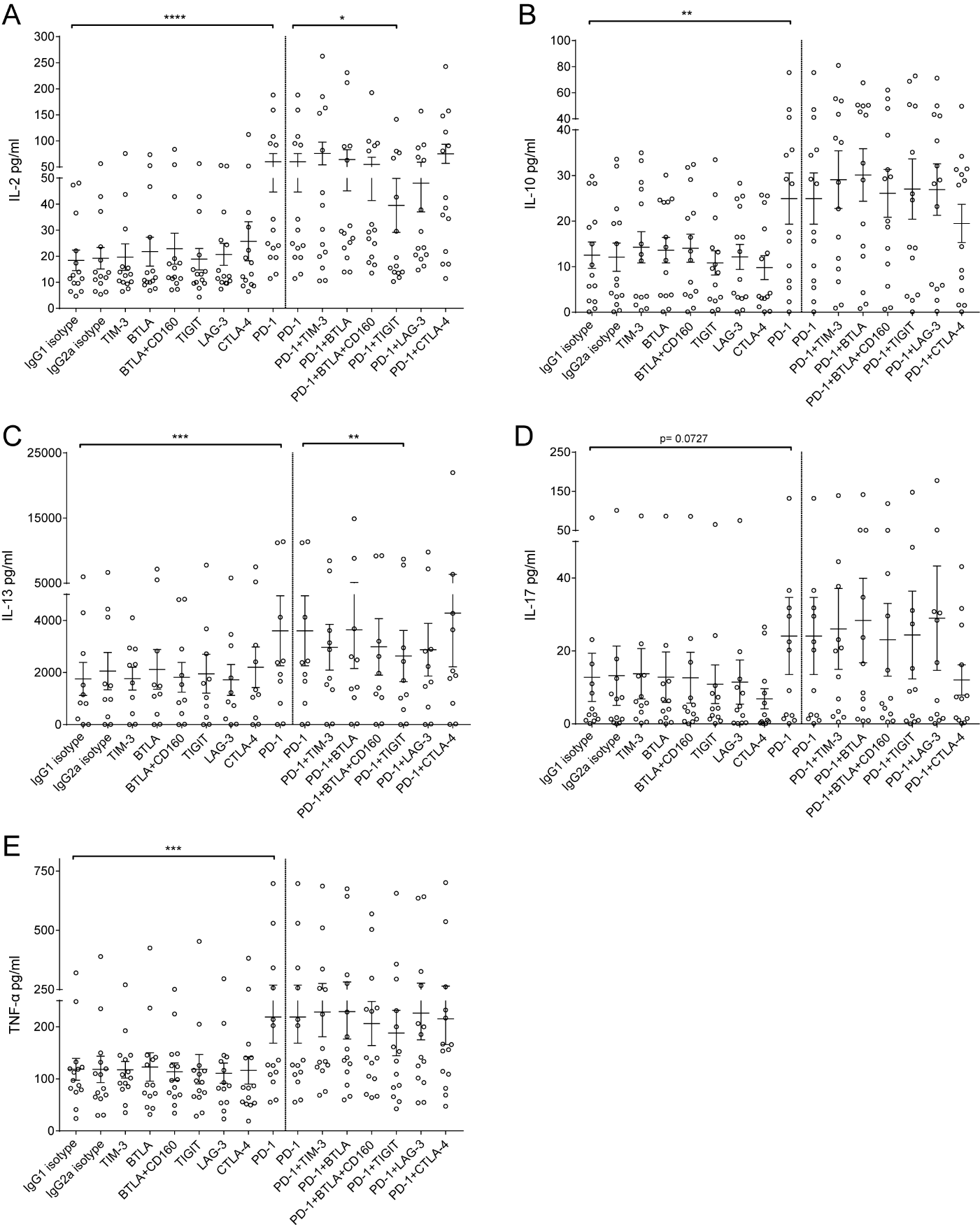
**Supplementary Figure 4.** **Effect of immune checkpoint inhibitors on the cytokine content in the culture supernatants.** T cells were co-cultured with 6x10^3^ mature allogeneic dendritic cells; culture supernatants were collected and analyzed by Luminex™-based multiplexing assays. The measured concentration of IL-2 **(A)**, IL-10 **(B)**, IL-13 **(C)**, IL-17 **(D)** and TNF-α **(E)** in the culture supernatant is shown (n=14, mean ± SEM).


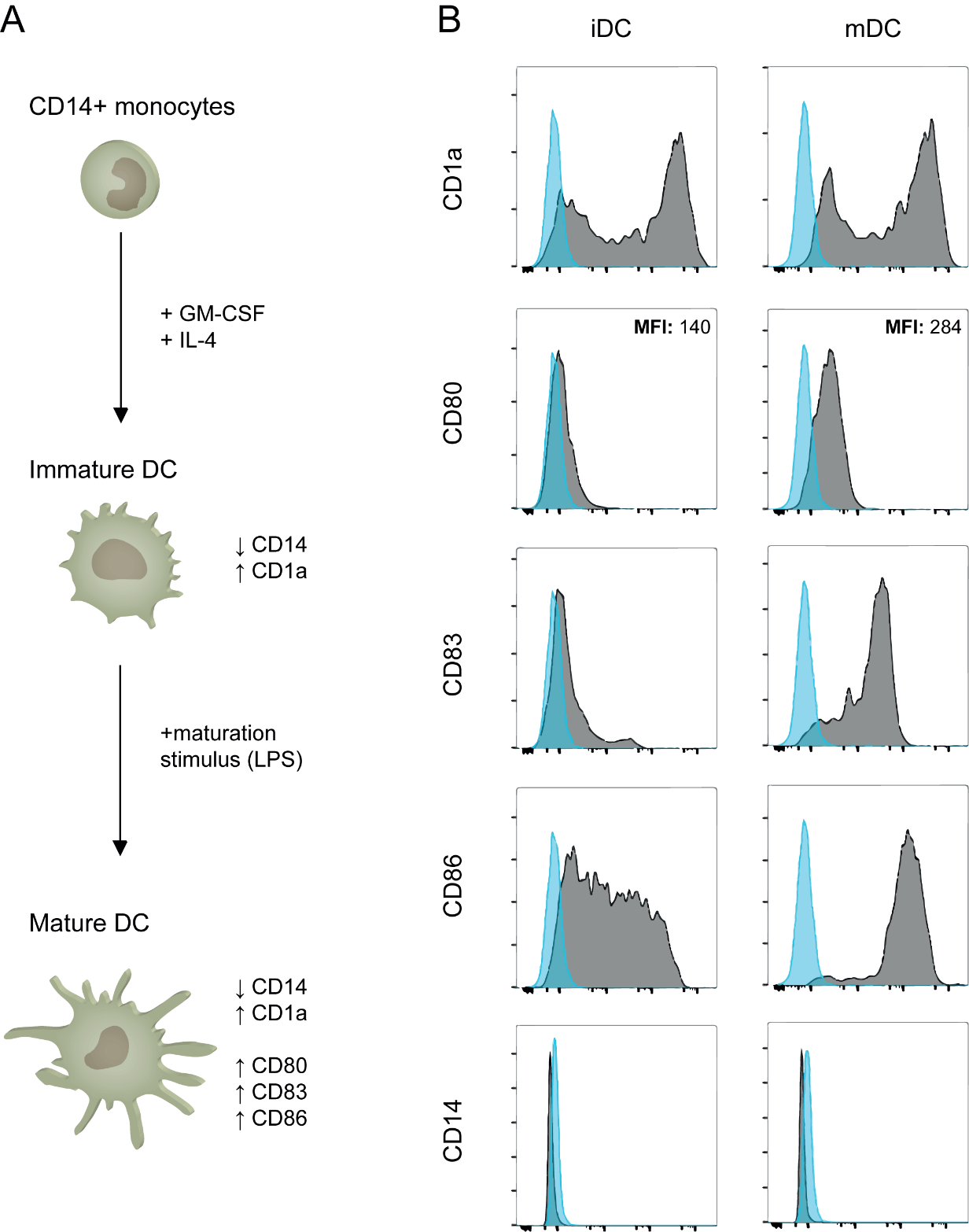


**Supplementary Figure 5.** **Dendritic cell maturation and control staining**. **A.** Scheme illustrating the differentiation of DC from human monocytes. **B.** Before co-culture with allogeneic T cells, immature DC (iDC) and mature DC (mDC) were stained for CD1a, CD80, CD83, CD86 and CD14; representative data from one experiment are shown. Isotype control antibody stainings are shown as blue histograms.
